# Determination of medical abortion success by women and community health volunteers in Nepal using a symptom checklist

**DOI:** 10.1186/s12884-018-1804-3

**Published:** 2018-05-11

**Authors:** Kathryn L. Andersen, Mary Fjerstad, Indira Basnett, Shailes Neupane, Valerie Acre, Sharad Sharma, Emily Jackson

**Affiliations:** 1 Ipas, 300 Market Street, Suite 200, Chapel Hill, NC 27516 USA; 2 San Diego, CA USA; 3 Kathmandu, Nepal; 4 Valley Research Group (VaRG), Lalitpur, Nepal; 5 Los Angeles, CA USA

**Keywords:** Medical abortion, Abortion, Nepal, Self-assessment, Community health volunteers

## Abstract

**Background:**

We sought to determine if female community health volunteers (FCHVs) and literate women in Nepal can accurately determine success of medical abortion (MA) using a symptom checklist, compared to experienced abortion providers.

**Methods:**

Women undergoing MA, and FCHVs, independently assessed the success of each woman’s abortion using an 8-question symptom checklist. Any answers in a red-shaded box indicated that the abortion may not have been successful. Women’s/FCHVs’ assessments were compared to experienced abortion providers using standard of care.

**Results:**

Women’s (*n* = 1153) self-assessment of MA success agreed with abortion providers’ determinations 85% of the time (positive predictive value = 90, 95% CI 88, 92); agreement between FCHVs and providers was 82% (positive predictive value = 90, 95% CI 88, 92). Of the 92 women (8%) requiring uterine evacuation with manual vacuum aspiration (*n* = 84, 7%) or medications (n = 8, 0.7%), 64% self-identified as needing additional care; FCHVs identified 61%. However, both women and FCHVs had difficulty recognizing that an answer in a red-shaded box indicated that the abortion may not have been successful. Of the 453 women with a red-shaded box marked, only 35% of women and 41% of FCHVs identified the need for additional care.

**Conclusion:**

Use of a checklist to determine MA success is a promising strategy, however further refinement of such a tool, particularly for low-literacy settings, is needed before widespread use.

## Background

Medical abortion (MA) using a combined regimen of mifepristone followed by misoprostol is highly effective with few complications when used to terminate pregnancies up to, and after, 70 days gestation [1–5]. In low-resource settings where access to health care and trained abortion providers is limited, MA can increase the availability of safe abortion, and decrease morbidity and mortality related to unsafe abortion [6]. In Nepal, which continues to face high abortion-related morbidity and mortality despite a liberal abortion law and government supported abortion services [7–10], provision of MA up to 63 days gestation has already been extended to include nurses and auxiliary nurse midwives trained as birth attendants, in addition to doctors [11, 12]. We evaluated the ability of a cadre of minimally-trained female community health volunteers (FCHVs) in Nepal and literate Nepali women to determine the success of MA with mifepristone and misoprostol using a checklist. Their assessments were compared to those made by comprehensive abortion care (CAC) trained providers using Nepal’s current standard of care.

## Methods

This study was conducted in two phases. In the previously published first phase, women and FCHVs used a toolkit consisting of a modified gestational dating wheel and a nine-point checklist of MA contraindications or cautions to assess women’s eligibility for MA, compared to experienced CAC-providers using standard of care in Nepal [13]. In the second phase, women and FCHVs used an eight-point checklist to determine if MA had been successful. For purposes of our study, success is defined as MA requiring no additional intervention, such as uterine aspiration or a repeat dose of misoprostol. Women and FCHV responses were compared to experienced CAC-providers. We report the second, success phase of the study in this paper.

### Study facilities

Seven study facilities, public and NGO, in six Nepali districts were purposively selected based on adequate abortion caseload to meet sample size requirements. All study facilities were located in urban centers, drawing patients from the surrounding rural areas.

### Study participants

Three thousand one hundred thirty-one literate women ≥16 years old (the age of consent in Nepal), with a positive pregnancy test, seeking safe abortion at a study facility were enrolled in the phase one, eligibility portion of this study. After completing the phase one study procedures, women received abortion by the method of her choice (uterine aspiration or MA with mifepristone and misoprostol) according to Nepali standard of care. All 1517 women who chose MA were invited to participate in the second, success phase of the study. These women were invited to return for a follow-up visit and evaluation in 2 weeks. Women received 400 Nepali rupees (approximately $5.20) and a month-supply of iron tablets for their participation in the success study. Those who agreed to participate in the success study but did not return for their 2-week follow-up visit were contacted by phone up to three times to encourage a return visit.

Nepal’s FCHVs receive a total of 18 days of training in maternal and child health, including training in early pregnancy identification with urine pregnancy tests; they are a key referral link in their communities to antenatal care, safe abortion, or family planning as appropriate [14]. Literate FCHVs were selected randomly from the six study districts and were posted at each study facility for one to 2 weeks during data collection. Participating FCHVs received a stipend of 3000 Nepali rupees (approximately $39) per week.

CAC-providers included doctors and staff nurses trained and registered with the Nepal Ministry of Health to provide CAC with manual vacuum aspiration and MA. All providers at the included facilities were eligible to participate.

All participants provided written, informed consent to participate.

### Sample size

Previous research indicates that > 90% of women receiving MA services will have a successful MA, and that the success checklist will have a 90% specificity to identify an unsuccessful MA [15]. We assumed a precision of 0.1 for both sensitivity (true positive rate) and specificity (true negative rate) of MA success determination, and a two-sided level of significance (α) of 0.5. Our calculated minimum sample size was 351 women. We continued to recruit beyond our minimum sample size until we had a minimum of 100 women for each public and 50 women for each private facility, and until we had a minimum of 50 women in each gestational age category, by weeks.

### Success checklist

**Fig. 1:**
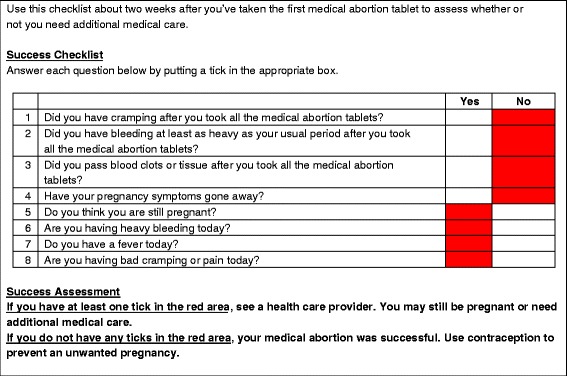
MA success checklist. The user answers each question. If any answer is in a red area, the woman may require additional care and should be seen by a provider

The success checklist (Fig. 1), adapted from Perriera et al. [16], is a series of eight questions assessing bleeding, cramping, and other symptoms following use of MA drugs, designed to determine if women have a continuing pregnancy or ongoing bleeding that would suggest the need for further care from a trained provider. In the real-world setting, if a woman or FCHV marks an answer in a red box on the checklist, the woman would be advised her abortion may not have been successful, and would be prompted to see a health care provider. If no red boxes are marked, the checklist indicates that the woman’s abortion was successful.

We tested the checklist for construct validity, and pretested it with Nepali women, FCHVs and CAC-providers. We edited the checklist following each round of pretesting; pretesting was considered complete when no further substantive comments were elicited.

### Study procedures

We collected data from September 2013 to March 2014. Each woman received a brief verbal orientation to the checklist, after which she used the checklist to self-determine whether or not her abortion had been successful. Demographic information and the woman’s assessment of the ease of use of the checklist were also collected. Women’s determinations of abortion success were sealed, and women were asked to not share their responses with the FCHV or CAC-provider they would subsequently meet. Next, an FCHV assessed the woman’s MA success using the checklist; her assessment was also sealed. Finally, a CAC-provider used local standard of care (typically a history and physical examination, with ultrasound available only when indicated) to assess abortion success. CAC-providers completed a brief questionnaire documenting their findings.

The Nepal Health Research Council and the Allendale Institutional Review Board in the United States reviewed this study for ethical considerations.

### Data analysis

To assess how well participants understood the success checklist, information entered into the checklist by women and FCHVs was compared to how they ultimately interpreted that information. The assessments of MA success made by women and by FCHVs were compared to those made by CAC-providers using 2 × 2 tables. Diagnostic test statistics (positive predictive value [PPV], negative predictive value [NPV], sensitivity [Sn] and specificity [Sp]) where women/FCHVs correctly identifying success was considered a positive test were generated.

## Results

Over three-quarters (76%) of the 1517 women enrolled in the phase one, eligibility portion of the study and who chose MA continued into the phase two, success portion of the study (*n* = 1153, Table 1). Women who continued into the success portion of the study were more likely to be married than women who did not participate in the second phase (97% vs 94%, *p* = 0.008); no other significant demographic differences were found (data not shown).

**Table 1:** Participant sociodemographic data

	Participants *n* = 1153
Age in years, mean (SD)	27.6 (5.4)
Pregnancies, mean (SD)	2.8 (1.3)
Some secondary school or higher, n (%)	1035 (90)
Married, n (%)	1113 (97)
Caste/Ethnicity, n (%)	
Disadvantaged Groups	351 (30)
Relatively Advantaged	190 (16)
Upper Caste Groups	612 (53)
Literacy, n (%)	1153 (100)

*SD* standard deviation

Disadvantaged groups includes dalit, disadvantaged janajaties, disadvantaged non-dalit Terai caste groups, and religious minorities

### Comprehension and ease of use of success checklist

Overall, 74% of women accurately interpreted their responses on the success checklist. All but two women with no red items marked (*n* = 698 of 700, 99.7%) correctly interpreted their checklist result as ‘successful’. However, of the 453 women who marked a red item, 295 (65%) did not identify that they may require additional care.

FCHVs interpreted the checklist correctly 100% of the time when no red boxes were marked as indicating a successful abortion, and misidentified women with a red box marked 59% of the time.

All women and FCHVs (100%) reported that the success checklist was easy to use. All women (100%) reported the checklist instructions were clear (yes/no), and 100% of FCHVs reported that the checklist instructions were either ‘very clear’ (54%) or ‘somewhat clear’ (46%).

### MA success assessment among women, FCHVs, and CAC-providers

When assessing MA success, women and providers agreed 85% of the time (Table 2); PPV was 90% (95% CI 88, 92). Providers identified 176 women (15%) as potentially requiring additional care. Of these, 84 (7%) received observation only; the remaining 92 (8%) received either uterine aspiration (*n* = 84, 7%) or readministration of medications (n = 8, 0.7%). Of the 92 women who required additional intervention, 64% (*n* = 59, Table 3) of women themselves perceived that additional care was needed using the success checklist. Of the 33 women who believed that their abortion was successful, six had marked a red item on the checklist. These women misinterpreted their response to the success checklist and should have identified that they needed additional care. The remaining 27 women (2% overall) had no red items marked, and their need for additional care would have been missed if using the checklist alone to determine abortion success.

**Table 2:** Women’s (*n* = 1153) and FCHVs’ (*n* = 159) assessments of MA success using the checklist are compared to CAC providers’ (*n* = 47) determinations based on standard of care

	Provider assessment MA successful		Provider assessment additional care needed		Total		PPV	NPV	Sn	Sp
	n	%^a^	n	%^a^	n	%^a^	% (95%, CI)	% (95% CI)	% (95% CI)	% (95% CI)
*Women’s Assessment*							90 (88, 92)	49 (41, 57)	92 (90, 93)	44 (36, 51)
MA successful	894	78	99	9	993	86				
Additional care needed	81	7	77	7	158	14				
Total	975	85	176	15	1151^b^	100				
*FCHVs’ Assessment*							90 (88, 92)	41 (34, 49)	89 (87, 91)	44 (36, 51)
MA successful	868	75	99	9	967	84				
Additional care neded	109	9	77	7	186	16				
Total	977	85	176	15	1153	100				

*PPV* positive predictive value, *NPV* negative predictive value, *Sn* sensitivity, *Sp* specificity

^a^Numbers may not add up to 100% due to rounding

^b^2 women missing self-assessment of success

**Table 3:** Women’s and FCHVs’ assessments of MA success using the success checklist for the 92 women that CAC-providers determined required additional intervention to complete their abortion (either uterine aspiration or readministration of medications)

	Women receiving additional intervention	
	n	Overall %
*Women’s Assessment*		
MA Successful	33	3
Additional care needed	59	5
Total	92	8
*FCHVs’ Assessment*		
MA Successful	36	3
Additional care needed	56	5
Total	92	8

Agreement for MA success between FCHVs and CAC-providers was 82%; PPV was 90% (95% CI 88, 92). Of the 92 women requiring additional intervention, FCHVs identified 61% (*n* = 56). FCHVs incorrectly identified four women who had a red item marked on the Success Checklist as having had a successful abortion; 32 women (3% overall) had no red items marked and their need for additional care would have been missed if the checklist alone was used.

## Discussion

We found that, when compared to CAC-trained providers using standard of care in Nepal to assess MA success, women using the success checklist agreed with CAC-providers 85% of the time, with a PPV of 90%. However these results must be interpreted with caution, given the significant difficulty that women had using the checklist. Although women reliably interpreted the checklist when no red items were marked (99.7%), more than half of the women who marked a red item (65%) did not realize they might need additional care, and should seek follow-up from a CAC-provider; FCHV results were similar. This is in contrast to earlier evidence indicating that women can accurately determine when their MA is successful using symptom-related questions. Indeed, in studies comparing women’s assessments of expulsion to those made by clinicians [16–19] and ultrasound [18], particularly when standardized questions are used [15, 16, 19], women have repeatedly proven themselves to be nearly as accurate as both. Despite their demonstrated ability to read, it is likely that women and FCHVs in our study did not understand either the checklist instructions, or the checklist items themselves. Indeed, ‘literacy’ in Nepal, where 43% of the population is illiterate [20], often indicates the ability to read and write at a basic level; overall reading comprehension in such a population is likely to be more limited and may have affected our findings. This may be different from ‘literacy’ in the highly literate industrialized nations (United States [14, 15], Scotland [18]) where earlier studies have been conducted. It is also possible that women and FCHVs, regardless of literacy level, required additional orientation to our success checklist to use it effectively. A recent study [21] evaluating the ability of community health workers in Ethiopia, India and South Africa to assess women’s eligibility for MA using a similar style of checklist oriented the workers to the checklist in a multi-day workshop. In contrast, the women and FCHVs in our study received a brief introduction, lasting only several minutes, to the checklist. Increasing comprehension of instructions and questions, reformatting of the checklist, moving away from the red = stop/green = go coloring scheme, which may not be suggestive enough in this setting, and use of newer technologies, such as a tablet computer, may make the checklist easier to use.

A small proportion of women who believed they had successful abortions based on the success checklist (2% of women’s assessments and 3% of FCHV’s assessments) were determined by CAC-providers to require additional intervention to complete their abortions. These women had marked no red items on their checklists. While it is possible that the checklist did not appropriately capture these as failures, it is also possible that the CAC-providers in our study were more conservative than expected in determining abortion success. The CAC-providers in our study did not utilize the success checklist when evaluating a patient, and instead relied on their own clinical practice and local standard of care. We found that CAC-providers in our study were more likely to provide additional treatment to women (8% of women received uterine evacuation with MVA or readministration of medications) than observed in other large MA efficacy trials up to 63 days gestation, where intervention rates are typically between 2 and 3% [22–24]. Given that an MA follow-up visit is optional in Nepal, it is possible that those women who returned for follow-up in our study were more likely to be experiencing complications, or were more anxious about experiencing complications. It is also possible, given that MA follow-up is not routine in Nepal, that CAC-providers may have been more inclined to intervene in women returning for interim care, a phenomenon that has been suggested in other studies [25]. We did not retrain the CAC-providers included in this study prior to study inception, which may have led them to be more conservative when determining MA success than currently recommended practice. As we considered CAC-providers to be the ‘gold standard’ for determining success, this is a limitation of our study.

More difficult than determining MA success, both for women and clinicians, has been the identification of those few MA cases where pregnancy is ongoing. As continuing pregnancy after MA is rare, few studies are adequately powered to assess this outcome [26], however a case-control study by Jackson et al. (2012) comparing 53 women with ongoing pregnancies following medical abortion to 53 controls with successful abortions, determined that up to a third of ongoing pregnancies are missed when women’s symptoms alone are used to assess completion [27]. While this would affect only a small absolute number of women, given potential teratogenicity of MA drugs and the need for timely uterine evacuation in these cases, the authors suggest that an objective measure of success, such as a urine pregnancy test, is still needed, in addition to report of women’s symptoms after MA drug use. Studies in both high [15] and low-resource settings [25] have explored combining a low sensitivity pregnancy test with women’s assessment of symptoms to determine MA success and identify rare, unrecognized continuing pregnancies. We did not include such a measure in this study, but this could be a consideration for future work.

Importantly, because of the high success rate of MA with mifepristone and misoprostol, WHO does not recommend routine follow-up after MA using the combined regimen. As provision of MA continues to be expanded to cadres of health care providers with less training [6], and perhaps even to women themselves, a reliable method of determining pregnancy expulsion and rare ongoing pregnancies, will be valuable.

## Conclusions

Although a promising strategy, both literate women and a cadre of minimally trained community health volunteers in Nepal had difficulty correctly using a symptom checklist to determine the success of MA. Refinement of such a tool, particularly for low literacy settings, is needed before widespread use.

## Abbreviations

CAC: Comprehensive abortion care
CI: Confidence interval
FCHV: Female community health volunteer
MA: Medical abortion
NGO: Non-governmental organization
NPV: Negative predictive value
PPV: Positive predictive value
Sn: Sensitivity
Sp: Specificity
